# Intramedullary nailing with and without cerclage in subtrochanteric fractures: an updated systematic review and meta-analysis

**DOI:** 10.1186/s12891-025-08999-w

**Published:** 2025-08-18

**Authors:** Ahmed Oun, Omar Abdelaziz, Islam Saeed Elhois, Mohamed Al-Badaineh, Karim Zinhom, George Jabrieh, Hamza S. Al-Himyari, Abdullah A. Nada

**Affiliations:** 1 Faculty of Medicine, Tanta University Hospital, Tanta University, Tanta, Egypt; 2 Faculty of Medicine, Alexandria University, Alexandria, Egypt; 3 Department of orthopedics, Qena General Hospital, Ministry of health and population, Qena, Egypt; 4 Jordan University of science and Technology, Irbid, Jordan; 5 Faculty of medicine, October 6 University, Giza, Egypt; 6 Faculty of medicine and Health sciences, Palestine Polytechnic University, Hebron, Palestine; 7 Department of orthopedics, Mabara Elmaadi Hospital, Ministry of health and population, Cairo, Egypt

**Keywords:** Subtrochanteric fractures, Femur, Intramedullary nail, Cerclage

## Abstract

**Background:**

Intramedullary nailing (IMN) is the gold standard for femoral subtrochanteric fractures (FSF) treatment due to their biomechanical advantages and minimally invasive nature. However, achieving proper reduction remains challenging in unstable patterns, leading to poor outcomes. Cerclage wiring, used alongside IMN, has shown potential in improving fracture stability and alignment, yet concerns regarding soft tissue damage persist. This systematic review and meta-analysis aims to evaluate the impact of cerclage wiring on clinical and radiological outcomes in FSF treatment.

**Methods:**

A comprehensive literature search was conducted for studies published up to the 3rd of March 2025 using PubMed, Scopus, Google Scholar, and Web of Science databases, focusing on randomized controlled trials and observational studies comparing intramedullary nailing with and without cerclage wiring in subtrochanteric fractures fixation. Eleven eligible studies, encompassing 658 patients, were included. The risk of bias was assessed using the Newcastle–Ottawa scale and meta-analytical techniques were employed to calculate pooled effect sizes and statistical significance.

**Results:**

Eleven retrospective studies were included revealing that cerclage wiring significantly increased operative time; *p* > 0.001, blood loss; *p* = 0.031, and better functional outcomes as union time significantly decreased; *p* = 0.026, significantly better Harris Hip Score; *p* = 0.012, while improving reduction quality. No significant differences were observed in non-union, infection, or mortality rates. Quality assessment using the Newcastle–Ottawa Scale indicated high quality studies.

**Conclusion:**

Cerclage wiring in conjunction with intramedullary nailing (IMN) for subtrochanteric fractures provides considerable advantages in our study in terms of the quality of fracture reduction, healing duration, and functional rehabilitation, especially in more complicated cases, even though it may lead to longer surgical times and greater blood loss.

**Supplementary Information:**

The online version contains supplementary material available at 10.1186/s12891-025-08999-w.

## Introduction

Femoral subtrochanteric fractures (FSF) are a type of fracture that occurs at the lesser trochanter and extends 5 cm distally, which can result in hip deformity, dysfunction, and limb disability [1]. FSF account for 5–20% of proximal femoral fractures [2, 3]. Subtrochanteric fractures can be associated with complications such as nonunion and implant failure. Proper surgical technique and minimal soft-tissue dissection are essential to reduce these risks [4].

Intramedullary nailing (IMN) is considered the gold standard for treating subtrochanteric femur fractures due to its biomechanical superiority and ability to provide stable constructs. It is preferred for its less invasive nature and effectiveness in achieving anatomical alignment [4, 5]. Anatomic reduction before fixation is key in these unstable patterns; however, due to the high degree of instability, achieving and maintaining good reduction alignment is not always feasible, resulting in poor outcomes such as nonunion, malunion, and implant failure [6, 7]. There is a need to provide additional support to IMN, and one of the reinforcement techniques is cerclage wiring. When used in conjunction with intramedullary nailing, cerclage wiring enhances fracture reduction and improves overall construct stability.

Despite numerous clinical trials evaluating the effectiveness of intramedullary cerclage wiring in subtrochanteric fractures, the results have been inconsistent. Studies indicate that cerclage wiring improves the quality of fracture reduction, reducing displacement and angulation, which are critical for successful healing [8, 9]. However, concerns about cerclage wiring include potential soft tissue damage and vascularity issues [10].

Therefore, high-quality evidence is needed to demonstrate the effect of cerclage wiring on clinical and radiological outcomes of surgical fixation of such fractures. This systematic review and meta-analysis aims to study the impact of cerclage wiring with IM nails on outcomes and complication rates in the treatment of subtrochanteric fractures.

## Methods

### Search strategy

A comprehensive literature search was conducted for studies published up to the 3rd of March 2025, utilizing PubMed, Scopus, Google Scholar, and Web of Science databases to retrieve studies comparing intramedullary nailing with and without cerclage wiring in subtrochanteric fractures.

The search strategy employed the following combination of terms: ((“Subtroch*” AND (“Femur” OR “femoral”) AND “Fracture” AND “Nail*” AND (“Cerclage” OR “Wir*” OR “Cable”)).This systematic review adhered to the Preferred Reporting Items for Systematic Reviewers and Meta-Analyses (PRISMA) guidelines (Supplementary file 1).

### Eligibility criteria

Inclusion criteria encompassed: (a) comparative studies of intramedullary nailing with and without cerclage wiring in subtrochanteric fractures; and (b) cohort studies and/or clinical trials on intramedullary nailing with and without cerclage wiring in non-metastatic subtrochanteric fractures. (c) adults above 18 years old.

Exclusion criteria comprised non-English language articles, review articles, case report, letter, or conference abstract, patients with pathological fractures.

### Study selection

Two reviewers manually screened the studies based on title and abstract then another two reviewers screened them based on full text, with subsequent verification by two independent reviewers to ensure accuracy and resolve disagreements.

### Data extraction

Four reviewers extracted relevant information from each included study, with subsequent verification by two independent reviewers to ensure accuracy.

Extracted data included year of publication, country of origin, study design, sample size, patient demographics, surgical technique (cerclage or non-cerclage), and outcomes (time to union, operation time, blood loss, length of hospital stay, follow up, Harris Hip Score (HHS), nonunion, reoperation rate, varus deformity, infections, leg length discrepancy, femoral neck angle, maximum cortical displacement, nail breakage, mortality rate and accuracy of fracture reduction which was determined modified from Baumgaertner et al. [11] as being good (both maximal cortical displacement < 4 mm and angulation < = 10 degrees), acceptable (either maximal cortical displacement < 4 mm or angulation < = 10 degrees), or poor (maximal cortical displacement > = 4 mm and angulation > 10 degrees).

### Quality assessment

The Newcastle–Ottawa Scale (NOS) was used to assess the quality of the included studies [12]. This tool evaluates three main domains: selection of study groups, comparability of groups, and outcome assessment.

Each study was scored based on these criteria, with higher scores indicating better quality. Two independent reviewers conducted the quality assessment, and any discrepancies were resolved through discussion.

### Data synthesis and statistical analysis

Standardized mean difference (SMD) with 95% Confidence Intervals (CIs) were pooled for continuous outcomes and odds ratio (OR) for dichotomous outcomes. A p-value > 0.05 was considered statistically significant for overall effect estimates. The DerSimonian and Laird random-effects model was applied. Heterogeneity was assessed using the Cochran Q test and I^2^ statistics, with *p* > 0.10 and I2 < 25% considered indicative of significant heterogeneity, following Cochrane’s guidelines. Statistical analyses were performed using Comprehensive Meta-Analysis (CMA) Version 4 (Biostat, Englewood, NJ, 2022).

## Results

### Study selection

**Fig. 1:**
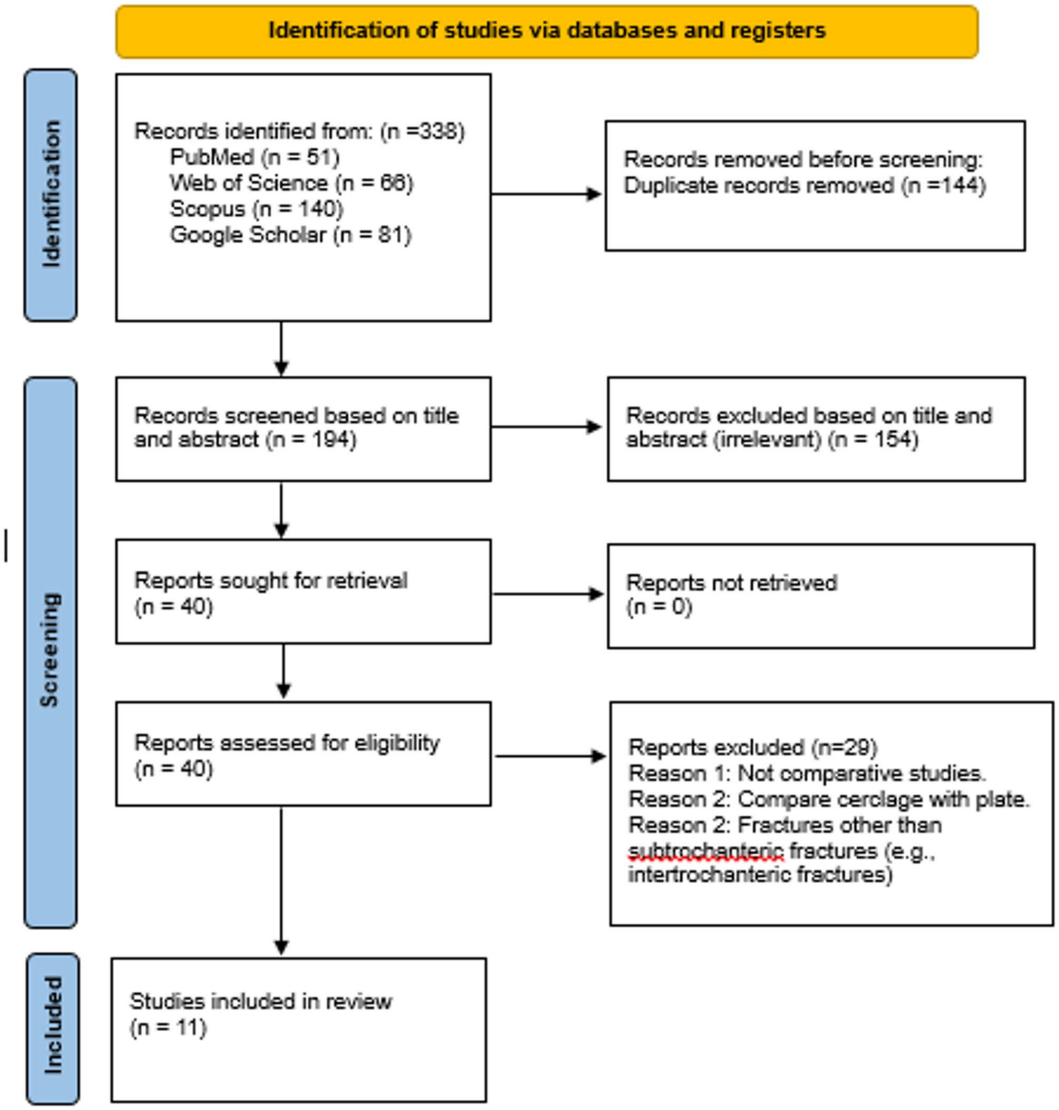
PRISMA flow diagram depicting the selection of the included articles

Initially, a total of 338 articles were identified. After the removal of 144 studies as duplicates, 194 articles were subjected to screening based on their titles and abstracts to assess eligibility. Of these studies, 40 articles were deemed suitable for full-text review. Upon full text screening, 29 studies were excluded due to reasons including non-English language, and lack of information on cerclage wiring and review articles. Ultimately, 11 articles [1, 2, 8, 13–20] were included in this review. All the studies were adjusted for comorbidities. Figure 1 illustrates the entire study selection process.

### Quality assessment

The methodological quality of the included studies was assessed using the Newcastle–Ottawa Scale (NOS) for cohort studies. All studies demonstrated adequate selection of exposed and non-exposed cohorts, with proper ascertainment of exposure, reducing selection bias.

The outcomes of interest were present nearly in all studies, ensuring a clear temporal relationship between exposure and outcomes. Most studies included a control group, providing a valid comparison. Outcome assessment was clearly reported across all studies, minimizing detection bias. Since all studies were observational, randomization and blinding were not applicable, possibly introducing performance bias. Despite these limitations, the included studies scored between 7 and 9 on the NOS scale, indicating high methodological quality (Table 1).

**Table 1:** Quality assessment of the studies

Study ID	1	2	3	4	5	6	7	8	9
Fatih İlker Can et al.	Yes	Yes	Yes	Yes	Yes	Yes	Yes	Yes	Yes
Dong Liu et al.	Yes	Yes	Yes	Yes	Yes	No	Yes	Yes	Yes
Mingjian Bei et al.	Yes	Yes	Yes	Yes	Yes	Yes	Yes	Yes	Yes
Yan-Hui Guo et al.	Yes	Yes	Yes	Yes	Yes	No	Yes	Yes	No
N. Bonfiglio et al.	Yes	Yes	Yes	Yes	Yes	Yes	Yes	Yes	Yes
Vivek Trikha et al.	Yes	Yes	Yes	Yes	Yes	Yes	Yes	Yes	Yes
Rajendra Annappa et al.	Yes	Yes	Yes	Yes	Yes	Yes	Yes	Yes	Yes
Ravindra Patil et al.	Yes	Yes	Yes	Yes	Yes	No	Yes	Yes	No
Sigrid fauconnier et al.	Yes	Yes	Yes	Yes	Yes	No	Yes	Yes	Yes
Mustafa Seyhan et al.	Yes	Yes	Yes	Yes	Yes	No	Yes	Yes	Yes
Jinpeng Gong et al.	Yes	Yes	Yes	Yes	Yes	No	Yes	Yes	Yes

### Characteristics of included studies

Table 2 presents the characteristics of the included studies, all of which were conducted retrospectively (Table 2). The analysis encompassed a total of 658 patients with subtrochanteric fractures, of whom 260 received cerclage wiring, while 398 did not. The mean age of patients in the studies ranges between 48.3 and 75.5 years with male predominance across them (Table 3).

**Table 2:** Summary of the included studies

Author	Year	Country	Duration of study	Type of study	Sample size	Number of patients in group 1 (cerclage group)	Number of patients in group 2 (non-cerclage group)
Fatih İlker Can et al.	2025	Turkey	2010–2018	Retrospective study	75	1-2 cables= 20//3-4 cables= 12	43
Dong Liu et al.	2022	China	2016–2019	Retrospective study	68	27	41
Mingjian Bei et al.	2024	China	2020–2021	Retrospective study	68	41	27
Yan-Hui Guo et al.	2024	China	2013–2021	Retrospective study	69	34	35
N. Bonfiglio et al.	2022	Italy	2016–2021	Retrospective study	80	33	47
Vivek Trikha et al.	2018	India	2012–2016	Retrospective study	48	21	27
Rajendra Annappa et al.	2020	India	2013– 2018	Retrospective study	55	14	41
Ravindra Patil et al.	2019	India	2016–2018	Retrospective study	34	19	15
Sigrid fauconnier et al.	2020	Belgium	2006–2016	Retrospective study	115	23	92
Mustafa Seyhan et al.	2012	Turkey	2005–2010	Retrospective study	33	11	22
Jinpeng Gong et al.	2016	China	2011–2013	Retrospective study	13	5	8

**Table 3:** Demographics and baseline characteristics

Authors	Mean Age (years) (mean ± SD)		Gender (M/F)		AO classification/seinsheimer classification	
	Cerclage	Non-cerclage	Cerclage	Non-cerclage	Cerclage	Non-cerclage
Fatih İlker Can et al.	48.3		M: 49F: 26		I: 6IIA: 7//IIB: 4//IIC: 10IIIA: 9//IIIB: 9IV: 15V: 15	
Dong Liu et al.	56.68± 41.48	57.66± 39.95	M: 19 F: 8	M: 30 F: 11	IIB:8//IIC:1//IIIA:4//IIIB:2//IV: 7//V: 5	IIB:13//IIC: 2//IIIA: 11//IIIB: 1//IV:9//V: 5
Mingjian Bei et al.	75.49±12.57	74.93±14.25	M: 18 F: 23	M: 9 F: 18	IIA:1//IIB: 6//IIC: 5//IIIA: 13//IIIB: 0//IV: 7//V: 9	IIA:4//IIB: 0//IIC: 0//IIIA:16//IIIB: 1//IV: 1//V: 5
Yan-Hui Guo et al.	58.21±16.66	53.66±19.27	M: 23 F: 11	M: 18 F: 17	II: 9//III:14//IV: 2//V: 10	II: 8//III: 11//IV: 2//V: 13
N. Bonfiglio et al.	74.4±18.6	73.9 ±19.2	M: 8 F: 25	M: 18 F: 29	31A3: 3//32A1: 11//32A2: 6//32B1: 6//32B1: 6//32C1: 4	31A3: 21//32A1: 13//32A2: 6//32B1: 2//32B1: 4//32C1: 1
Vivek Trikha et al.	49.19 ± 18.16	50.52 ± 18.03	M: 9 F: 12	M: 14 F: 13	32 A1.1: 4//32 A2.1: 2//32 B1.1: 10//32 C1.1: 5	32 A1.1: 5//32 A2.1: 6//32 B1.1: 7//32 C1.1: 9
Rajendra Annappa et al.	56.4		NA	NA	NA	NA
Ravindra Patil et al.	48.9 ± 19.7	53.5 ± 19.3	M: 9 F: 6	M: 12 F: 7	NA	NA
Sigrid fauconnier et al.	62.91±23.497	67.17±21.953	M: 12 F: 11	M: 45 F: 47	NA	NA
Mustafa Seyhan et al.	55.82±19.34	55.32±23.61	M: 8 F: 3	M: 8 F: 14	IIA: 0//IIB: 0//IIC: 1//IIIA: 3//IIIB:3//IV: 1//V: 1	IIA: 2//IIB: 3//IIC: 4//IIIA: 4//IIIB: 2//IV: 2//v: 5
Jinpeng Gong et al.	63.4 ±11.39	59.88 ±11.42	NA	NA	32 A1.1: 0//32 A2.1: 0//32 B1.1: 1//32 C1.1: 4	32 A1.1: 3//32 A2.1: 0//32 B1.1: 4//32 C1.1: 1

Fracture patterns were classified using either the Seinsheimer or AO classification systems. The group that received cerclage wiring exhibited a higher incidence of complex fractures (Seinsheimer types III–V and AO 32B1/C1), whereas the group without cerclage wiring predominantly had simple or intermediate fractures (Seinsheimer II and AO 31A3/32A1). Within the cerclage group, Seinsheimer Type V was the most frequent (ranging from 18.5 to 28.6%), followed by Type III (ranging from 14.8 to 40.0%), with 32B1 fractures being the most common (up to 47.6% in one study). 32C1 (comminuted) fractures constituted 12.1–23.8%. While in the non-cerclage group, Seinsheimer Type III was the most prevalent (ranging from 26.8 to 59.3%), with fewer Type V fractures (12.2–38.2%). 31A3 (simple trochanteric) fractures were common (44.6% in one study), and 32A1 fractures ranged from 18.5 to 27.6% (Table 3).

### Outcomes

**Fig. 2:**
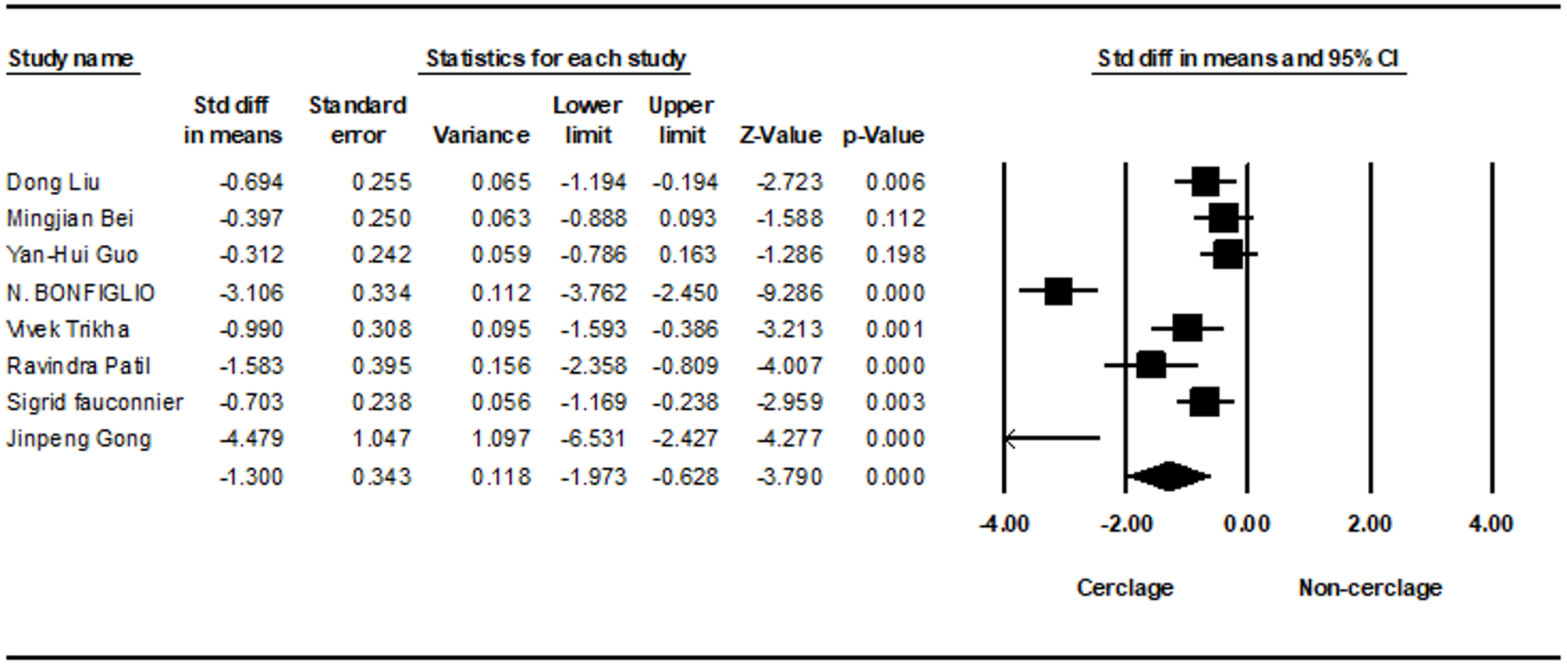
Forest plot comparing operation time between cerclage vs. no cerclage

Continuous outcomes were analyzed using SMD while dichotomous outcomes were in OR as follows:

A meta-analysis of eight studies was performed to compare operative time between intramedullary nailing with and without cerclage for subtrochanteric fracture fixation. The pooled effect size demonstrated a statistically significant increase in operative time associated with cerclage fixation (− 1.300; 95% CI −1.973 to − 0.628; *p* = 0.001), with high heterogeneity (I² = 89.91%) (Fig. 2).

**Fig. 3:**
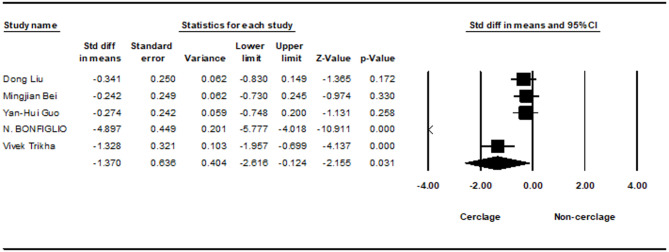
Forest plot comparing blood loss between cerclage vs. no cerclage

Similarly, a meta-analysis of five studies evaluated intraoperative blood loss between the two groups. The results indicated a statistically significant increase in blood loss in the cerclage group (− 1.370; 95% CI −2.616 to − 0.124; *p* = 0.031), with substantial heterogeneity (I² = 95.97%) (Fig. 3).

**Fig. 4:**
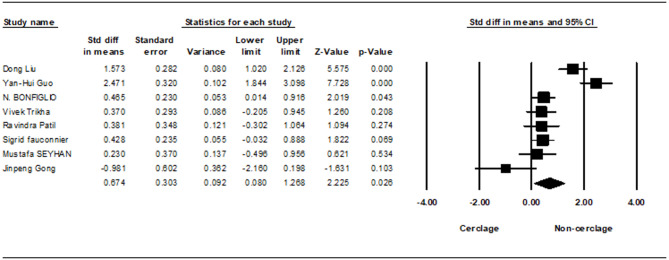
Forest plot comparing union time between cerclage vs. no cerclage

Union time was assessed across seven studies, revealing a statistically significant increase in union time in the non-cerclage group (0.674; 95% CI 0.080–1.268; *p* = 0.026), with high heterogeneity (I² = 87.03%) (Fig. 4).

A meta-analysis of five studies compared the HHS between the two groups, demonstrating a statistically significant improvement in functional outcomes associated with cerclage fixation (− 0.466; 95% CI −0.827 to − 0.104; *p* = 0.012), with moderate heterogeneity (I² = 51.10%) (Supplementary file).

Postoperative reduction quality was evaluated using Baumgartner’s criteria. Six studies compared the achievement of good reduction, demonstrating a statistically significant improvement with cerclage (3.918; 95% CI 1.940 to 7.913; *p* = 0.001, I² = 37.26%). Similarly, cerclage was associated with significantly higher rates of acceptable reduction (0.324; 95% CI 0.191 to 0.549; *p* = 0.001, I² = 0%). Conversely, non-cerclage fixation was associated with a significantly higher rate of poor reduction (0.285; 95% CI 0.113–0.719; *p* = 0.008, I² = 0%) (Supplementary file).

Hospital length of stay was assessed in four studies, showing no statistically significant difference between groups (− 0.280; 95% CI −0.744 to 1.184; *p* = 0.236, I² = 73.34%) (Supplementary file). Similarly, no significant difference was observed in follow-up duration between groups (− 0.174; 95% CI −0.429 to 0.082; *p* = 0.183, I² = 0%) (Supplementary file) (Tables 4, 5, 6).

**Table 4:** Clinical outcomes of cerclage wiring vs. no cerclage in subtrochanteric fractures

Author	Operation time (mean ± SD) (Min)		Blood loss (mean ± SD) (ml)		Hospital stay (mean ± SD) (days)	
	Cerclage	Non cerclage	Cerclage	Non cerclage	Cerclage	Non cerclage
Fatih İlker Can et al.	NA	NA	NA	NA	NA	NA
Dong Liu et al.	124.01±35.28	102.05±29.04	172.59±83.79	145.73±75.50	9.52±2.01	8.22±1.97
Mingjian Bei et al.	86.22±32.09	73.70±30.59	331.71±164.60	292.59±156.71	5.71±2.96	6.04±2.82
Yan-Hui Guo et al.	192.66±75.85	170±69.57	366.66±309.57	300±154.60	13.37±4.26	14 ±4.64
N. Bonfiglio et al.	254.7±−80.2	84.7±24.6	224.4 ±37.8	87.3±18.3	18.7 ±9.4	12.4 ±8.3
Vivek Trikha et al.	104.47±14.53	87.59±18.77	227.5± 52.5	150 ±62.5	NA	NA
Rajendra Annappa et al.	NA	NA	NA	NA	NA	NA
Ravindra Patil et al.	96.74 ± 24.53	62.95 ± 16.35	median 180	median 120	NA	NA
Sigrid fauconnier et al.	120± 39	92 ± 40	NA	NA	NA	NA
Mustafa Seyhan et al.	NA	NA	NA	NA	NA	NA
Jinpeng Gong et al.	125.4± 6.5	92.25± 7.87	NA	NA	NA	NA

**Table 5:** Clinical outcomes of cerclage wiring vs. no cerclage in subtrochanteric fractures

Author	Follow-up (mean ± SD) (months		Union time (mean ± SD) (months)		Harris Hip Score	
	Cerclage	Non cerclage	Cerclage	Non cerclage	Cerclage	Non cerclage
Fatih İlker Can et al.	12.33		5.4 in 1-2 cables//5.7 in 3-4 cables	7.3	NA	NA
Dong Liu et al.	19.44±5.53	17.58±4.58	3.56±0.64	4.82±0.89	75.59±8.88	76.29±9.27
Mingjian Bei et al.	25.02±6.58	24.14±6.44	NA	NA	79.17±14.65	74.00±14.63
Yan-Hui Guo et al.	NA	NA	5.17±1.55	9±1.55	93.83±3.48	90.33±5.41
N. Bonfiglio et al.	16.1±4.2	15.4±3.6	3.4 ±2.1	4.2 ±1.4	NA	NA
Vivek Trikha et al.	NA	NA	17.14±3.29 weeks= 3.95 ± 0.76 months	18.15±2.13 weeks= 4.18 ± 0.49 months	NA	NA
Rajendra Annappa et al.	NA	NA	NA	NA	NA	NA
Ravindra Patil et al.	NA	NA	14.5±3.29 weeks= 3.34 ± 0.76 months	15.6±2.13 weeks= 3.59 ± 0.49 months	89.25±1.01	88.12±3.13
Sigrid fauconnier et al.	NA	NA	6.91 ± 2.84	8.62 ± 4.23	NA	NA
Mustafa Seyhan et al.	24.18±5.78	26.09±10.88	19±8 weeks= 4.37 ± 1.84 months	21±9 weeks= 4.83 ± 2.07 months	89.91±7.78	83.05±6.56
Jinpeng Gong et al.	NA	NA	21.4 ± 2.3 weeks= 4.92 ± 0.53 months	19.13 ± 2.30 weeks= 4.40 ± 0.53 months	89.85±3.5

**Table 6:** Reduction achieved of cerclage wiring vs. no cerclage in subtrochanteric fractures

Author	Reduction achieved					
	Cerclage			Non cerclage		
	Good	Acceptable	Poor	Good	Acceptable	Poor
Fatih İlker Can et al.	NA	NA	NA	NA	NA	NA
Dong Liu et al.	22	5	0	24	12	5
Mingjian Bei et al.	27	14	0	9	16	2
Yan-Hui Guo et al.	31			24		
N. Bonfiglio et al.	23	8	2	13	27	7
Vivek Trikha et al.	20	1	0	20	3	4
Rajendra Annappa et al.	9	3	2	9	23	9
Ravindra Patil et al.	14	1	0	15	2	2
Sigrid fauconnier et al.	NA	NA	NA	NA	NA	NA
Mustafa Seyhan et al.	NA	NA	NA	NA	NA	NA
Jinpeng Gong et al.	NA	NA	NA	NA	NA	NA

**Fig. 5:**
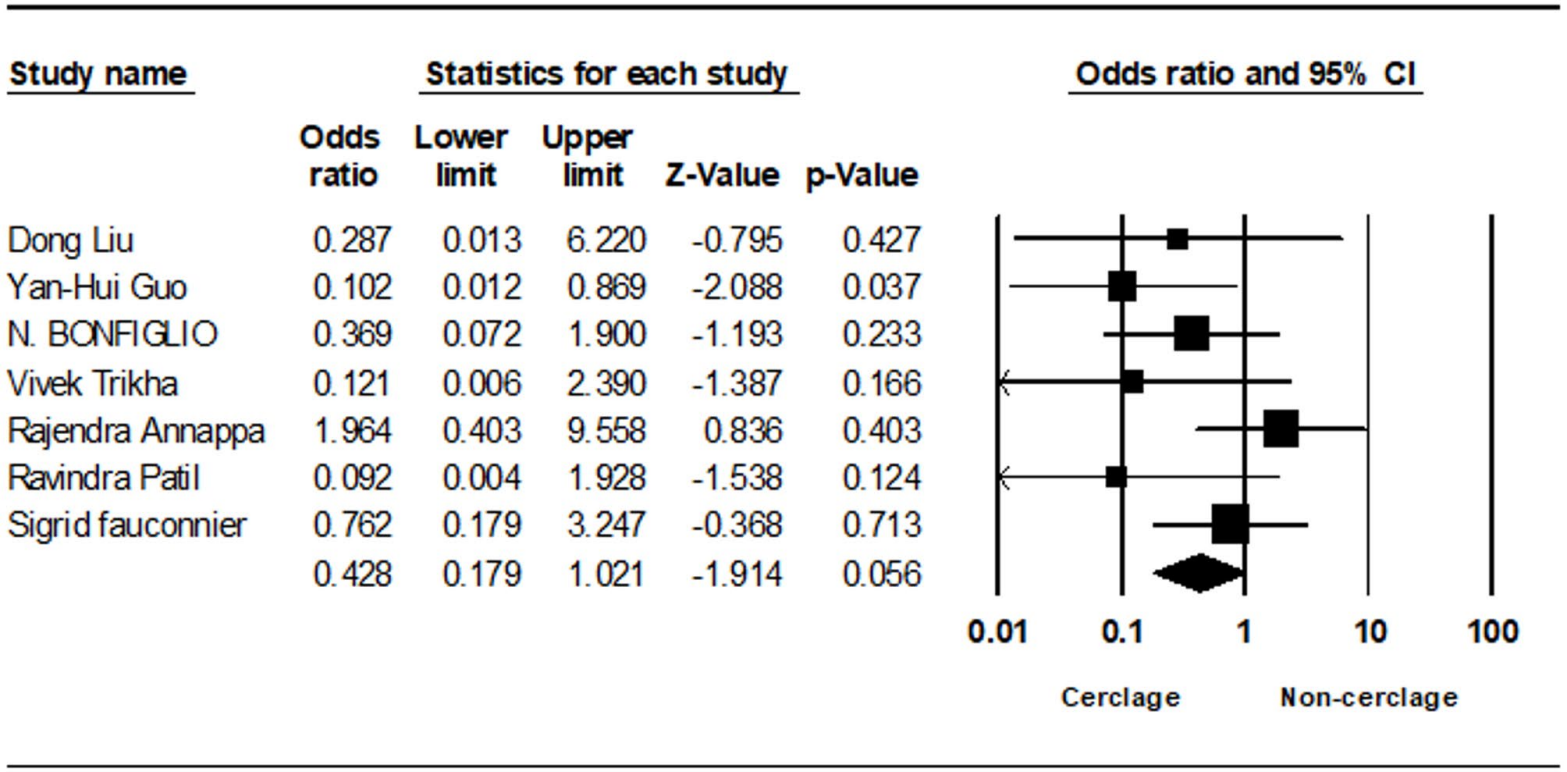
Forest plot comparing nonunion between cerclage vs. no cerclage

**Fig. 6:**
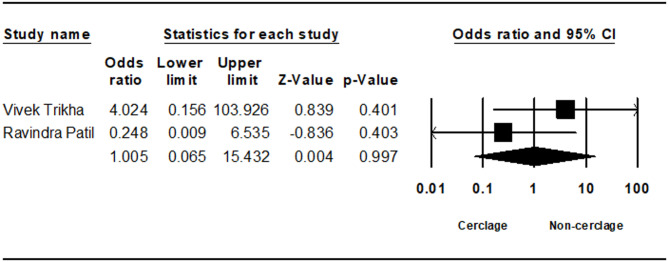
Forest plot comparing delayed union between cerclage vs. no cerclage

Seven studies analyzed non-union rates, showing no statistically significant difference, although non-cerclage fixation trended towards a higher incidence (0.428; 95% CI 0.179 to 1.021; *p* = 0.056, I² = 20.99%) (Fig. 5). Delayed union, evaluated in two studies, also demonstrated no significant difference (1.005; 95% CI 0.065 to 15.432; *p* = 0.997, I² = 28.71%) (Fig. 6).

Implant-related complications were assessed, including nail breakage in three studies, which showed a statistically significant increase in the non-cerclage group (0.135; 95% CI 0.023–0.777; *p* = 0.025, I² = 0%) (Supplementary file).

**Fig. 7:**
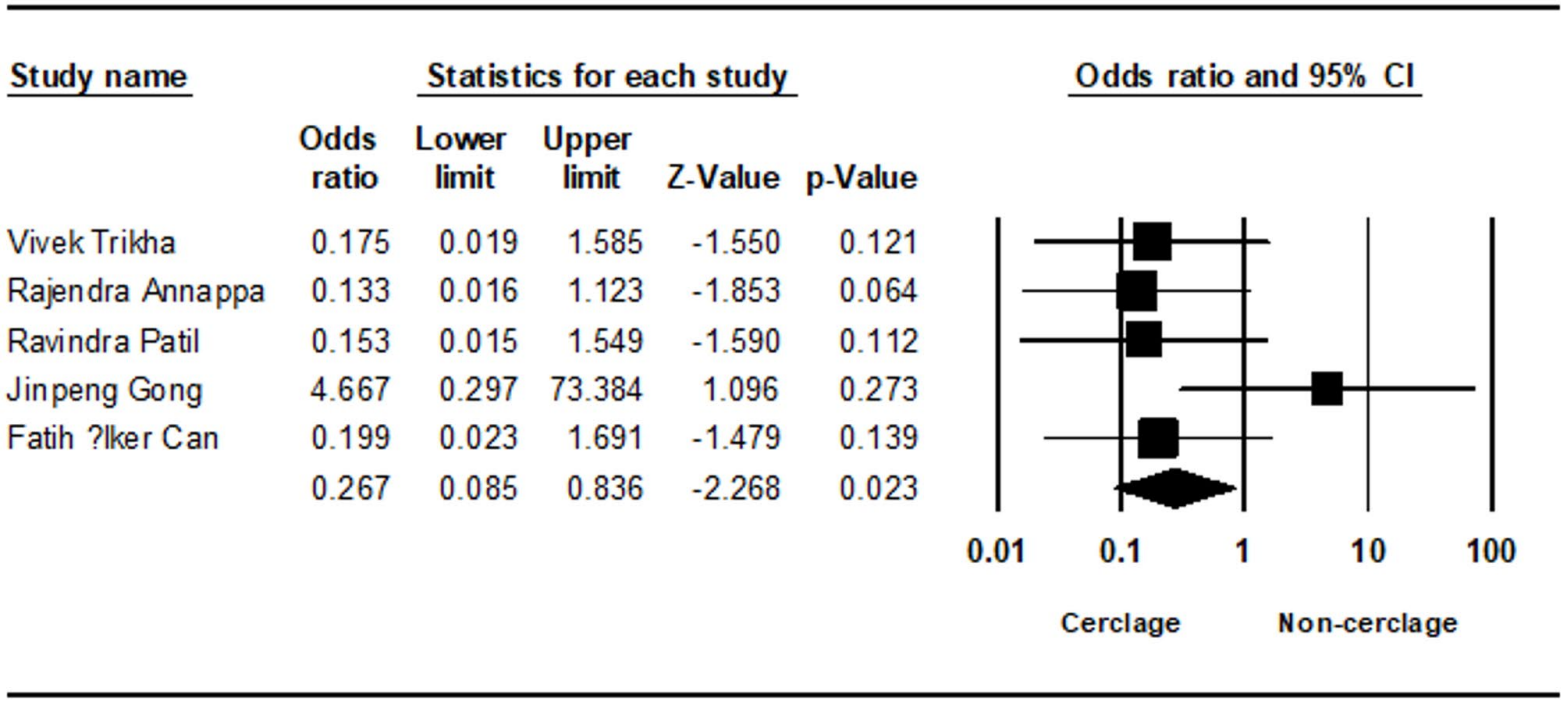
Forest plot comparing varus deformity between cerclage vs. no cerclage

Varus deformity, assessed in five studies, was also significantly higher in the non-cerclage group (0.267; 95% CI 0.085 to 0.836; *p* = 0.023, I² = 19.73%) (Fig. 7). Similarly, angulation, evaluated in two studies, was significantly more pronounced in the non-cerclage group (0.768; 95% CI 0.316 to 1.219; *p* = 0.001, I² = 0%) (Supplementary file).

Femoral neck angle was compared across four studies, demonstrating a significantly higher angle in the cerclage group (− 0.477; 95% CI −0.746 to − 0.209; *p* = 0.001, I² = 0%) (Supplementary file). Leg length discrepancy, assessed in three studies, showed no statistically significant difference (0.497; 95% CI 0.109 to 2.276; *p* = 0.368, I² = 7.49%) (Supplementary file).

However, maximum cortical displacement was significantly greater in the non-cerclage group (0.868; 95% CI 0.539 to 1.197; *p* = 0.001, I² = 0%) (Supplementary file).

Superficial wound infection rates, analyzed in three studies, did not differ significantly between groups, though there was a trend towards higher rates in the non-cerclage group (1.531; 95% CI 0.257–9.123; *p* = 0.640, I² = 0%) (Supplementary file). Similarly, deep infection rates, examined in three studies, were not significantly different (2.236; 95% CI 0.535–1.102; *p* = 0.270, I² = 0%) (Supplementary file).

**Fig. 8:**
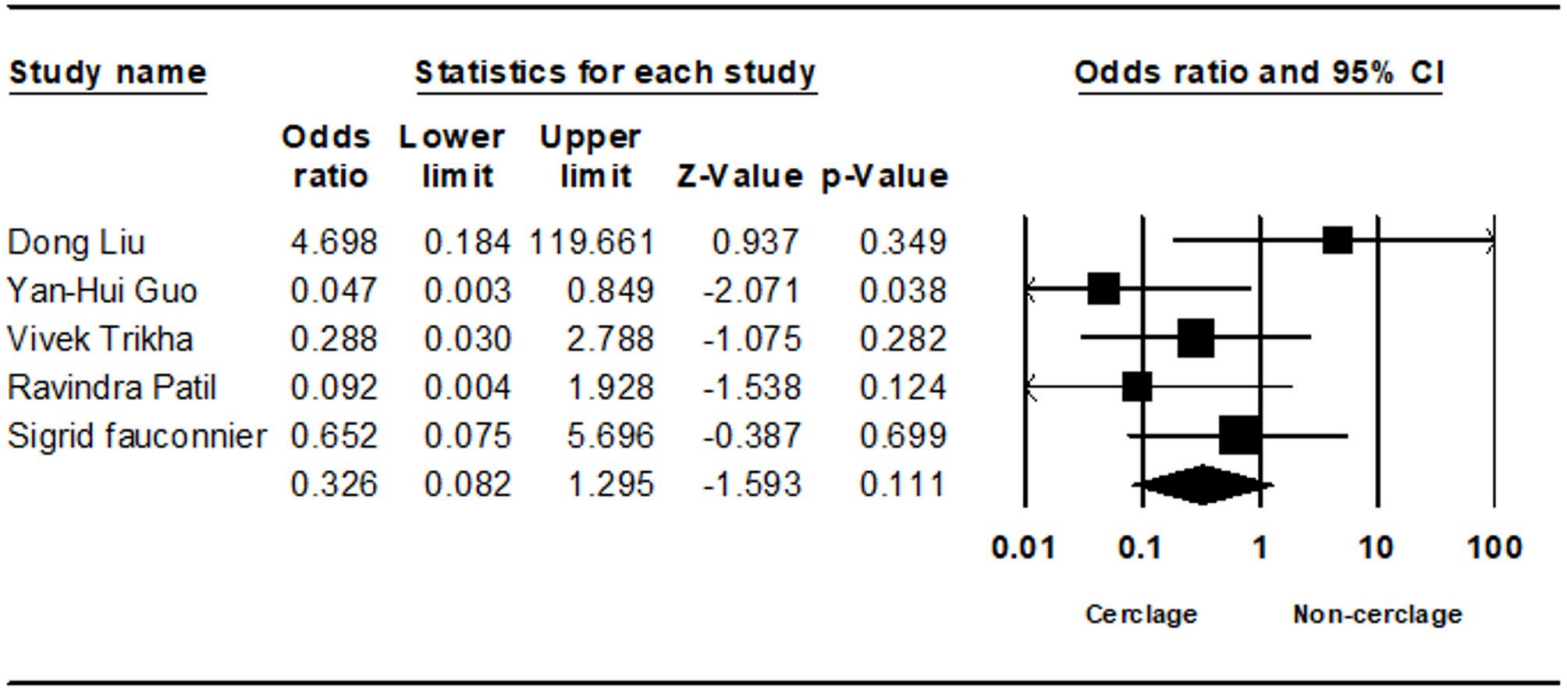
Forest plot comparing reoperation rate between cerclage vs. no cerclage

**Fig. 9:**
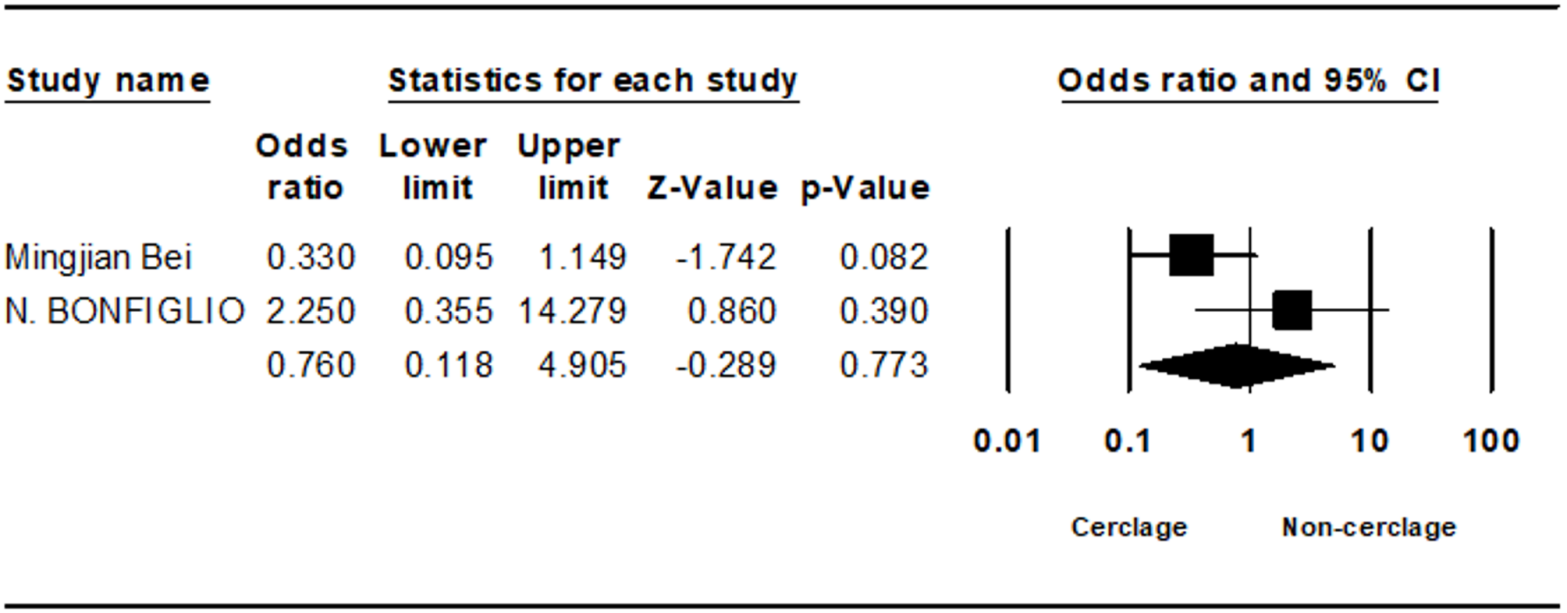
Forest plot comparing mortality rate between cerclage vs. no cerclage

Reoperation rates, analyzed in five studies, showed no statistically significant difference between groups, with a trend towards higher rates in the non-cerclage group (0.326; 95% CI 0.082 to 1.295; *p* = 0.111, I² = 25.95%) (Fig. 8). Mortality rates, assessed in two studies, also showed no statistically significant difference (0.894; 95% CI 0.274 to 2.911; *p* = 0.852, I² = 18.52%) (Fig. 9) (Tables 7, 8, 9, 10).

**Table 7:** Complications of cerclage wiring in subtrochanteric fractures

Author	Cerclage					
	Superficial SSI	Deep SSI	Non-union	Reoperation	Varus angulation	Mortality
Fatih İlker Can et al.	NA	NA	NA	NA	1	NA
Dong Liu et al.	1	0	0	1	NA	NA
Mingjian Bei et al.	NA	NA	NA	NA	2.35±1.78 angle loss	5
Yan-Hui Guo et al.	NA	NA	1	NA	NA	NA
N. Bonfiglio et al.	2	2	2	NA	NA	3
Vivek Trikha et al.	0	0	0	1	1	NA
Rajendra Annappa et al.	NA	2	3	NA	1	NA
Ravindra Patil et al.	1	NA	NA	0	1	NA
Sigrid fauconnier et al.	0	0	3 of 12	1	NA	NA
Mustafa Seyhan et al.	NA	NA	NA	NA	NA	NA
Jinpeng Gong et al.	NA	NA	NA	NA	2	NA

**Table 8:** Complications of cerclage wiring in subtrochanteric fractures

Author	Cerclage					
	Delayed union	Angulation	Leg length discrepancy (LLD)	Maximum cortical displacement	femoral neck angle	Nail breakage
Fatih İlker Can et al.	NA	NA	NA	NA	NA	NA
Dong Liu et al.	NA	NA	NA	NA	127.44±2.08	NA
Mingjian Bei et al.	NA	NA	NA	NA	NA	NA
Yan-Hui Guo et al.	NA	NA	NA	NA	NA	NA
N. Bonfiglio et al.	NA	NA	NA	NA	NA	NA
Vivek Trikha et al.	1	3±2.5	1	2±1.25	134.4±2.39	NA
Rajendra Annappa et al.	NA	NA	NA	NA	NA	NA
Ravindra Patil et al.	NA	3± 2.5	0	1.9 +- 1.25	130.1.+ 2.2	NA
Sigrid fauconnier et al.	NA	NA	NA	1.30 ± 2.62	128.6 ± 6.7	NA
Mustafa Seyhan et al.	NA	NA	NA	NA	NA	NA
Jinpeng Gong et al.	NA	NA	2	NA	NA	NA

**Table 9:** Complications of Non cerclage wiring in subtrochanteric fractures

Author	Non cerclage					
	Superficial SSI	Deep SSI	Non-union	Reoperation	Varus angulation	Mortality
Fatih İlker Can et al.	NA	NA	NA	NA	9	NA
Dong Liu et al.	1	0	2	0	NA	NA
Mingjian Bei et al.	NA	NA	NA	NA	6.88±2.86 angle loss	8
Yan-Hui Guo et al.	NA	NA	8	8 (22.9)	NA	NA
N. Bonfiglio et al.	1	0	7	NA	NA	2
Vivek Trikha et al.	0	0	4	4	6	NA
Rajendra Annappa et al.	1	3	5	NA	15	NA
Ravindra Patil et al.	NA	NA	3	3	4	NA
Sigrid fauconnier et al.	NA	2	14 of 46	6	NA	NA
Mustafa Seyhan et al.	NA	NA	NA	NA	NA	NA
Jinpeng Gong et al.	NA	NA	NA	NA	1	NA

**Table 10:** Complications of Non cerclage wiring in subtrochanteric fractures

Author	Non cerclage					
	Delayed union	Angulation	Leg length discrepancy (LLD)	Maximum cortical displacement	femoral neck angle	Nail breakage
Fatih İlker Can et al.	NA	NA	NA	NA	NA	NA
Dong Liu et al.	NA	NA	NA	NA	126.46±2.89	NA
Mingjian Bei et al.	NA	NA	NA	NA	NA	NA
Yan-Hui Guo et al.	NA	NA	NA	NA	NA	NA
N. Bonfiglio et al.	NA	NA	NA	NA	NA	8
Vivek Trikha et al.	NA	5± 3	4	3.5±2	133.02±3.68	3
Rajendra Annappa et al.	NA	NA	NA	NA	NA	NA
Ravindra Patil et al.	1	5.5±3.48	2	4.1±2.31	127.7 ± 2.5	2
Sigrid fauconnier et al.	NA	NA	NA	9.04± 11.9	126.5± 5.4	NA
Mustafa Seyhan et al.	NA	NA	NA	NA	NA	NA
Jinpeng Gong et al.	NA	NA	2 (<1 cm)	NA	NA	NA

Overall, the heterogeneity was observed among the included studies, likely attributable to variations in surgical techniques, patient demographics, and methodological differences across studies.

## Discussion

This systematic review evaluates the efficacy of adding cerclage wiring to IMN in subtrochanteric fractures. The addition of cerclage wiring was associated with significantly increased operative time (*p* = 0.001) and intraoperative blood loss (*p* = 0.031), attributable to the technical demands of soft tissue dissection and fracture manipulation required for anatomical alignment prior to nail insertion [9, 21].

Similar perioperative increases were noted by Hoskins et al., Kennedy et al., and Kim et al., reinforcing the procedural complexity [9, 21, 22]. Trikha et al. observed an average 28-minute increase in surgical duration with percutaneous cerclage but noted reduced soft tissue damage [14]. Mehta et al. reported prolonged operative time and greater blood loss in type III fractures managed with cerclage, though hospital length of stay remained unaffected (*p* = 0.236) [23]. These intraoperative demands must be balanced against patient frailty and urgency of care, as emphasized by Kasha et al. [24].

Despite increased surgical burden, cerclage wiring demonstrated clear benefits in fracture healing. The meta-analysis revealed a significantly shorter union time in the cerclage group (*p* = 0.026) and a trend toward reduced non-union rates in comparison to IMN alone (*p* = 0.056). Additionally, cerclage wiring achieved significantly higher rates of good and acceptable reductions (*p* = 0.001 for both), while non-cerclage approaches were associated with more frequent poor reductions (*p* = 0.008), as defined by Baumgartner’s criteria.

Biomechanical studies by Husemoglu et al. and Müller et al. attribute these improvements to enhanced construct stability and reduced interfragmentary movement, facilitating primary bone healing [25, 26].

Fracture pattern appears to influence the utility of cerclage. Guo et al. and Kang et al. reported shorter union times (18.6 vs. 22.4 weeks) in complex fracture types (Seinsheimer III–V, AO 32B1/C1) when cerclage was used [13, 27].

Similarly, Tomás et al. and Hantouly et al. documented improved union rates in unstable fractures [28, 29]. In contrast, Knauf et al. found that IMN alone achieved satisfactory reduction in 85% of simpler AO 31 A fractures, suggesting that cerclage may confer limited benefit in less complex scenarios [30]. The high heterogeneity in union time (I² = 87.03%) likely reflects variability in fracture classification, surgical technique, and postoperative care, underscoring the need for standardized treatment protocols.

Functionally, cerclage wiring was associated with a modest but statistically significant improvement in HHS (*p* = 0.012). This may be attributable to more accurate anatomical reduction and earlier mobilization, particularly in elderly populations [31–33].

Panteli et al. reported an average HHS of 82 in patients treated with cerclage compared to 76 in the non-cerclage group at six months postoperatively [33]. However, moderate heterogeneity (I² = 51.10%) suggests functional outcomes are influenced by patient-related variables, such as age, comorbidities, and rehabilitation regimens.

Complication profiles also differed between the groups. The non-cerclage cohort demonstrated significantly higher incidences of implant-related complications, including nail fractures (*p* = 0.025), varus deformity (*p* = 0.023), malalignment (*p* = 0.001), and cortical displacement (*p* = 0.001).

These findings are consistent with biomechanical limitations of IMN alone in managing unstable fractures, as described by Perren and Wang et al. [34, 35]. Cerclage wiring mitigated varus collapse (5% vs. 15%), and Afsari et al. advocated for supplementary fixation to optimize femoral neck alignment and reduce malreduction risk [36, 37]. Although infection rates, reoperation rates, and mortality did not differ significantly, Kasha et al. noted a trend toward fewer reoperations in the cerclage group (3% vs. 8%), possibly reflecting improved construct durability and perioperative management [24].

Overall, this meta-analysis suggests that cerclage wiring, despite its increased intraoperative demands, enhances reduction quality, accelerates healing, and improves functional outcomes—particularly in complex subtrochanteric fractures (Seinsheimer III–V, AO 32B/C). Evidence from Hoskins et al., Kim et al., and Hantouly et al. supports its selective application in cases necessitating precise fracture alignment [9, 22, 29].

Conversely, for less severe patterns (Seinsheimer II, AO 31A3), IMN alone may suffice, reducing surgical morbidity [16, 30]. Treatment strategies should be individualized based on fracture complexity, patient age, and comorbidities, as emphasized by Codesido et al. and Kim et al. [22, 32].

The significant heterogeneity observed across several outcomes (e.g., I² = 89.91% for operative time) reflects the limitations of retrospective data, including inter-surgeon variability and patient heterogeneity. This underscores the urgent need for prospective, randomized controlled trials employing consistent fracture classifications, standardized surgical techniques, and long-term follow-up. Additionally, future investigations should incorporate cost-effectiveness analyses, particularly for resource-limited settings, as proposed by Guo et al. [13].

This study has several limitations. All included studies were retrospective, introducing potential selection bias despite high methodological quality (Newcastle-Ottawa Scale scores: 7–9). Considerable heterogeneity (e.g., I² = 89.91% for operative time; 87.03% for union time) limits the generalizability of pooled estimates.

Moreover, limited sample sizes for certain outcomes (e.g., delayed union and mortality) resulted in wide confidence intervals and reduced statistical power. Long-term functional outcomes beyond HHS were sparsely reported, and follow-up durations were variable and often insufficient.

Finally, unadjusted confounding factors, such as surgeon experience, implant selection, and rehabilitation protocols, may have influenced outcomes and warrant consideration in future research.

## Conclusions

Cerclage wiring in conjunction with intramedullary nailing (IMN) for subtrochanteric fractures provides considerable advantages in our study in terms of the quality of fracture reduction, healing duration, and functional rehabilitation, especially in more complicated cases, even though it may lead to longer surgical times and greater blood loss.

## Supplementary Information

Supplementary Material 1

## Data Availability

Availability of data and materialAll data generated and analyzed throughout this study were included either in this article or its supplementary information file.

## Abbreviations

N/A: Not applicable
SD: Standard deviation

## References

[1] Bei M, Xiao Y, Xu Y, Chen Y, Cao Q, Zhao C, Li N, Tian F, Yang M, Wu X. Enhanced outcomes in femoral subtrochanteric fractures using long INTERTAN nails with titanium cable cerclage: A retrospective analysis. Med Sci Monit. 2024;30:e944383. 10.12659/MSM.944383. 39039768 10.12659/MSM.944383 PMC11299482

[2] Liu D, Liu HZ, Ma ML, Zhou N, Wang H. The clinical efficacy of minimally invasive Clamp-Assisted reduction and open reduction with wire cerclage for unstable subtrochanteric fractures. J Healthc Eng. 2022;2022(5340504). 10.1155/2022/5340504. 10.1155/2022/5340504 PMC8808220 35126929

[3] Loizou C, McNamara I, Ahmed K, Pryor G, Parker M. Classification of subtrochanteric femoral fractures. Injury. 2010;41(7):739–45. 10.1016/j.injury.2010.02.018. 20394921 10.1016/j.injury.2010.02.018

[4] Garrison I, Domingue G, Honeycutt M. Subtrochanteric femur fractures: current review of management. EFORT Open Reviews. 2021;6:145–51. 10.1302/2058-5241.6.200048. 33828858 10.1302/2058-5241.6.200048 PMC8022017

[5] Inchaustegui M, Ruiz K, Gonzalez M, Pretell-Mazzini J. Surgical management of metastatic pathologic subtrochanteric fractures. JBJS Rev. 2023;11. 10.2106/JBJS.RVW.22.00232. 10.2106/JBJS.RVW.22.00232 37141383

[6] Haidukewych GJ, Israel TA, Berry DJ. Reverse obliquity fractures of the intertrochanteric region of the femur. J Bone Joint Surg. 2001;83(5):643–50. 10.2106/00004623-200105000-00001. 11379732 10.2106/00004623-200105000-00001

[7] Kilinc BE, Oc Y, Kara A, Erturer RE. The effect of the cerclage wire in the treatment of subtrochanteric femur fracture with the long proximal femoral nail: a review of 52 cases. Int J Surg. 2018;56:250–5. 10.1016/j.ijsu.2018.06.035. 29960077 10.1016/j.ijsu.2018.06.035

[8] Fauconnier S, Van Lieshout M, Victor J. Evaluation of cerclage wiring in the treatment of subtrochanteric fractures. Acta Orthop Belg. 2020;86(1):28–32. 32490770

[9] Hoskins W, Bingham R, Joseph S, Liew D, Love D, Bucknill A, Oppy A, Griffin X. Subtrochanteric fracture: the effect of cerclage wire on fracture reduction and outcome. Injury. 2015;46(10):1992–5. 10.1016/j.injury.2015.07.001. 26264881 10.1016/j.injury.2015.07.001

[10] Hoskins W, McDonald L, Spelman T, Bingham R. Subtrochanteric femur fractures treated with femoral nail: the effect of cerclage wire augmentation on complications, fracture union, and reduction: A systematic review and Meta-Analysis of comparative studies. J Orthop Trauma. 2021;36:e142–51. 10.1097/BOT.0000000000002266. 10.1097/BOT.0000000000002266 34510127

[11] Baumgaertner MR, Curtin SL, Lindskog DM, et al. The value of the tip-apex distance in predicting failure of fixation of peritrochanteric fractures of the hip. J Bone Joint Surg Am. 1995;77:1058–64. 7608228 10.2106/00004623-199507000-00012

[12] Wells GA, Shea B, O’Connell D, Peterson J, Welch V, Losos M, Tugwell P. The Newcastle–Ottawa scale (NOS) for assessing the quality of nonrandomized studies in meta-analyses. Ott Hosp Res Inst. 2011. 10.13140/RG.2.1.1158.4485.

[13] Guo YH, Song ZL, Zheng HY, Gao J, Lin YY, Liu Z, Li LH. Intramedullary nailing for irreducible spiral subtrochanteric fractures: A comparison of cerclage and non-cerclage wiring. Chin J Traumatol. 2024;27(5):305–10. 10.1016/j.cjtee.2024.03.011. 38641468 10.1016/j.cjtee.2024.03.011 PMC11401497

[14] Trikha V, Das S, Agrawal P, Kumar Dhaka MA. Role of percutaneous cerclage wire in the management of subtrochanteric fractures treated with intramedullary nails. Chin J Traumatol. 2018;21(1):42–9. 10.1016/j.cjtee.2018.01.001. 29426797 10.1016/j.cjtee.2018.01.001 PMC5835546

[15] Seyhan M, Unay K, Sener N. Comparison of reduction methods in intramedullary nailing of subtrochanteric femoral fractures. Acta Orthop Traumatol Turc. 2012;46(2):113–9. 10.3944/AOTT.2012.2639. 22491436 10.3944/AOTT.2012.2639

[16] Can Fİ, Gültaç E, Kılınç RM, Kılınç CY. The effect of cable fixation on union time in subtrochanteric femur fractures treated with cephalomedullary nailing. Northwest Med J. 2025;5(1):1–8. 10.54307/2025.NWMJ.140.

[17] Bonfiglio N, Smimmo A, Carosini A, Perna A, Ruberto P, Minutillo F, De Santis V, Malerba G. Subtrochanteric fractures in elderly people: functional and radiographic outcomes after intramedullary locked nail fixation with or without cerclage. Eur Rev Med Pharmacol Sci. 2022;26(1 Suppl):127–37. 10.26355/eurrev_202211_30292. 36448870 10.26355/eurrev_202211_30292

[18] Gong J, Yang Y, Liu P, Nie X, Li R, Wu J, Sun Q, Ge W, Cai M. PFNA with reduction assisted with pointed clamp and cable cerclagefor select subtrochanteric fractures of the femur. 2016;9:2961–8.

[19] Annappa R, Kamath S, Krishnamurthy S, Mallya S, Kamath K, Suresh P. Does cerclage wiring with intramedullary nailing in subtrochanteric fractures improve the final outcome? Medico-Legal Update. 20; 2020. 10.37506/mlu.v20i3.1417

[20] Patil R, Modi S, Bhalla A, Rajoli S, Kumar R, Ghelani G. Effect of encerclage wiring with intermedullary nailing in subtrochanteric fractures of femur. Indian J Orthop Surg (IJOS). 2019;5(1):35–41. 10.18231/j.ijos.2019.007.

[21] Kennedy MT, Mitra A, Hierlihy TG, Harty JA, Reidy D, Dolan M. Subtrochanteric hip fractures treated with cerclage cables and long cephalomedullary nails: a review of 17 consecutive cases over 2 years. Injury. 2011;42(11):1317–21. 10.1016/j.injury.2011.03.023. 21497812 10.1016/j.injury.2011.03.023

[22] Kim CH, Yoon YC, Kang KT. The effect of cerclage wiring with intramedullary nail surgery in proximal femoral fracture: a systematic review and meta-analysis. Eur J Trauma Emerg Surg. 2022;48(6):4761–74. 10.1007/s00068-022-02003-z. 35618854 10.1007/s00068-022-02003-z

[23] Mehta NJ, Goldsmith T, Lacey A, Reddy G, Selvaratnam V, Ramakrishnan M. Outcomes of intramedullary nailing with cerclage wiring in subtrochanteric femoral fractures. Strateg Trauma Limb Reconstr. 2019;14(1):29–33. 10.5005/jp-journals-10080-1423. 10.5005/jp-journals-10080-1423 PMC7001599 32559265

[24] Kasha S, Yalamanchili RK. Management of subtrochanteric fractures by nail osteosynthesis: a review of tips and tricks. Int Orthop. 2020;44(4):645–53. 10.1007/s00264-019-04404-z. 31529139 10.1007/s00264-019-04404-z

[25] Husemoglu RB, Havıtçıoğlu H. Biomechanical comparison of different subtrochanteric bone fracture angles in cerclage wiring: finite element study. J Med Innov Technol. 2021;3(2):35–9. 10.51934/jomit.1052710.

[26] Müller T, Topp T, Kühne CA, Gebhart G, Ruchholtz S, Zettl R. The benefit of wire cerclage stabilisation of the medial hinge in intramedullary nailing for the treatment of subtrochanteric femoral fractures: a Biomechanical study. Int Orthop. 2011;35(8):1237–43. 10.1007/s00264-010-1204-4. 21258791 10.1007/s00264-010-1204-4 PMC3167430

[27] Kang SJ, Bao FL, Huang DS, Jiang T, Hu YM, Li JM, Liu T. Percutaneous cerclage wiring combined with cephalomedullary nailing for irreducible subtrochanteric fractures. Orthop Surg. 2021;13(6):1899–911. 10.1111/os.13144. 34435729 10.1111/os.13144 PMC8523774

[28] Tomás J, Teixidor J, Batalla L, Pacha D, Cortina J. Subtrochanteric fractures: treatment with cerclage wire and long intramedullary nail. J Orthop Trauma. 2013;27(7):e157–60. 10.1097/BOT.0b013e31826fc03f. 22932753 10.1097/BOT.0b013e31826fc03f

[29] Hantouly AT, Salameh M, Toubasi AA, Salman LA, Alzobi O, Ahmed AF, Ahmed G. The role of cerclage wiring in the management of subtrochanteric and reverse oblique intertrochanteric fractures: a meta-analysis of comparative studies. Eur J Orthop Surg Traumatol. 2023;33(4):739–49. 10.1007/s00590-022-03240-z. 35377073 10.1007/s00590-022-03240-z PMC10125946

[30] Knauf T, Eschbach D, Buecking B, Knobe M, Barthel J, Rascher K, Ruchholtz S, Aigner R, Schoeneberg C, On Behalf Of The Registry For German Trauma Dgu. Open reduction in subtrochanteric femur fractures is not accompanied by a higher rate of complications. Med (Kaunas). 2021;57(7):659. 10.3390/medicina57070659. 10.3390/medicina57070659 PMC8305416 34199013

[31] Bukhari RR, et al. A prospective study of clinico-radiological outcome assessment in proximal femoral fractures treated with proximal femoral nail. Eur J Mol Clin Med. 2020;7(07). https://ejmcm.com/article_3509.html. [Open Access]

[32] Codesido P, Mejía A, Riego J, Ojeda-Thies C. Subtrochanteric fractures in elderly people treated with intramedullary fixation: quality of life and complications following open reduction and cerclage wiring versus closed reduction. Arch Orthop Trauma Surg. 2017;137(8):1077–85. 10.1007/s00402-017-2722-y. 28555367 10.1007/s00402-017-2722-y

[33] Panteli M, Vun JSH, West RM, Howard A, Pountos I, Giannoudis PV. Surgical site infection following intramedullary nailing of subtrochanteric femoral fractures. J Clin Med. 2021;10(15):3331. 10.3390/jcm10153331. 34362123 10.3390/jcm10153331 PMC8347680

[34] Perren SM. Evolution of the internal fixation of long bone fractures. The scientific basis of biological internal fixation: choosing a new balance between stability and biology. J Bone Joint Surg Br. 2002;84(8):1093–110. 10.1302/0301-620x.84b8.13752. 12463652 10.1302/0301-620x.84b8.13752

[35] Wang TH, Chuang HC, Kuan FC, Et. Role of open cerclage wiring in patients with comminuted fractures of the femoral shaft treated with intramedullary nails. J Orthop Surg Res. 2021;16:480. 10.1186/s13018-021-02633-w. 34364374 10.1186/s13018-021-02633-w PMC8348994

[36] Afsari A, Liporace F, Lindvall E, Infante A Jr, Sagi HC, Haidukewych GJ. Clamp-assisted reduction of high subtrochanteric fractures of the femur: surgical technique. J Bone Joint Surg Am. 2010;92(Suppl 1 Pt 2):217–25. 10.2106/JBJS.J.00158. 20844177 10.2106/JBJS.J.00158

[37] Xie H, Xie L, Wang J, Chen C, Zhang C, Zheng W. Intramedullary versus extramedullary fixation for the treatment of subtrochanteric fracture: A systematic review and meta-analysis. Int J Surg. 2019;63:43–57. 10.1016/j.ijsu.2019.01.021. 30735845 10.1016/j.ijsu.2019.01.021

